# Evaluation of Vaso-occlusive Crises in United States Sickle Cell Disease Patients: A Retrospective Claims-based Study

**DOI:** 10.36469/9667

**Published:** 2019-05-03

**Authors:** Nirmish Shah, Menaka Bhor, Lin Xie, Steve Arcona, Rashid Halloway, Jincy Paulose, Huseyin Yuce

**Affiliations:** 1 Duke University; 2 Novartis Pharmaceuticals Corporation; 3 SIMR, Inc; 4 New York City College of Technology, CUNY

**Keywords:** erythropoiesis, iron, red cells

## Abstract

Sickle cell disease (SCD) is a life-threatening vascular disease that burdens affected persons physically. SCD related vaso-occlusive crises (VOCs) are one of the primary causes of morbidity and mortality. Our objective was to examine the epidemiology of pain crises and the relationship between pain crises and major acute complications among SCD patients. Using the Medicaid Analytic Extracts from 2009-2013, patients with SCD were selected and the first clinical claim indicating SCD during the identification period was defined as the index date. Patients were required to have continuous Medicaid enrollment for ≥6 months pre- and 12 months post-index period. Clinical outcomes included mortality, inpatient pain crises, and complications. Cox regressions were applied to examine the relationship between pain crises and deaths or acute complications, respectively. A total of 20 909 patients were included with a mean age of 17.9 years. The rate of VOC events in 100 person-years was 142.20 for adults and 53.91 for pediatric patients. Patients with VOCs were associated with a higher risk for death (hazard ratio=1.56; 95% confidence interval: [1.19-2.05]) or acute complications including acute chest syndrome, stroke, pulmonary embolism, splenic sequestration, and pulmonary hypertension. SCD patients have a substantial burden of disease-related complications. This study suggests that inpatient vaso-occlusive crisis is a key risk factor for acute complications.

## Background

Sickle cell disease (SCD) is a life-threatening vascular disorder, which early on progresses into a systemic disease.^1,2^ SCD can affect various races and occurs more often among African American and Hispanic- American births.^3^ SCD affects ~100,000 Americans.^4^

Vaso-occlusive crises (VOCs), also known as acute sickle cell pain crises, are the most common, painful complication of the disease and the main reason why patients seek medical care in hospitals. Vaso-occlusive events are driven by endothelium damage/dysfunction and inflammation, which can then lead to vascular damage. SCD patients’ erythrocytes have the propensity to change into crescent shapes with abnormal adhesive properties, which increase interactions with white blood cells, platelets, endothelial cells, and extracellular matrix proteins. All these abnormal interactions accelerate the development of clinical vasculopathy and promote cerebrovascular and pulmonary vascular diseases. VOC significantly affects patients’ quality of life and often requires emergency care.^5^ Several diary studies of SCD patients have reported high rates of pain as well as an intense severity of pain during study days.^6–8^ VOC is the leading cause of hospitalization among SCD patients,^9^ and health care utilization (HRU) among SCD patients remains high due to patients’ difficulty with acute pain management and the lack of new effective therapies.^10^

SCD-related complications are associated with early mortality and high morbidity, and VOC is often a precursor of these complications.^11^ In the 1990s, the majority of deaths among SCD patients occurred prior to the third year of life and mainly due to infections, acute chest syndrome (ACS), and splenic sequestration crises.^12^ Vaccination advancements have largely decreased early childhood mortality among SCD patients, but life expectancy has not improved much for adult SCD patients over the last era.^13^ While death among SCD patients is often unexpected, and the direct cause of death unclear, cardiopulmonary diseases including ACS, stroke, and pulmonary hypertension remain the most commonly reported complications associated with death.^14,15^ Due to the common mechanism of vaso-occlusion and SCD-related complications, VOC events have been found to precede other acute complications such as ACS in up to 80% of cases.^16^ Most patients initially seek acute care for a VOC episode; however, as the crisis event is now recognized as a primary risk factor for additional life-threatening complications,^11^ VOC has become a marker of disease severity and an indicator of premature mortality in the modern patient cohort.^17^

Though pediatric and adult SCD patients experience many similar symptoms and management issues, SCD complications and disease outcomes usually differ by age.^18^ Hydroxyurea has improved health outcomes in both populations. Health outcomes have improved and stabilized in the pediatric population, but the adult population has experienced an increasing mortality rate: there is a great unmet need for new therapies and an understanding of the underlying disease factors contributing to the increased mortality. Further, hydroxyurea will not fully resolve the symptoms of a sizeable proportion of patients in both age cohorts, leading to an unmet need in both populations.^19^ To understand the burden and impact of VOC among SCD patients in a real-world setting, this study evaluated the rate of complications and associations between VOC and life-threatening complications.

## Methods

### Data Source

This was a retrospective, observational cohort study using US Medicaid databases. The study period ranged from January 1st, 2009 through December 31st, 2013, with the identification period between July 1st, 2009 and December 31st, 2012 so that every patient would have a ≥6-month baseline and ≥1-year follow-up period.

The Medicaid Analytic Extracts (MAX) data system contains extensive individual-level information on the characteristics of Medicaid enrollees in all 50 states and the District of Columbia as well as the services used during a calendar year. Specifically, MAX consists of one personal summary file and four claims files that provide fee-for-service claims, managed care encounter data, and premium payments. The study included fee-for-service patients from all available states and Managed Care enrollees who resided in 14 states with relatively complete data: Arizona, California, Indiana, Kansas, Kentucky, Minnesota, Nebraska, New Jersey, New Mexico, New York, Oregon, Tennessee, Texas, and Virginia. Service use among Managed Care enrollees is captured in encounter data. Medicaid enrollees who had dual edibility with Medicare are not included in this study due to incomplete information in the MAX data.

### Patient Selection

Patients were included in the study if they had ≥1 clinical claim code related to SCD (International Classification of Diseases, 9th Revision, Clinical Modification [ICD-9-CM] codes 282.41, 282.42, 282.60- 282.69) during the identification period (July 1st, 2009 to December 31st, 2012). Patients were required to have continuous health plan enrollment with medical and pharmacy benefits during the 6 months before the index date (baseline period) and 1 year after the index date and to not be enrolled in a clinical trial during the study period (identified using ICD-9-CM code V70.7). The first observed SCD-related clinical code during the identification period was designated as the index date. Patient data were assessed until the earliest of disenrollment, death, or the end of the study period.

### Baseline Measures

Variables during the 6-month baseline period were measured. Sociodemographic variables including age, sex, race, and US geographic region (Northeast, North Central, South, and West) were flagged during the patient selection process. The baseline Charlson comorbidity index (CCI) score was calculated based on the most updated version of the CCI.^20^ Baseline individual comorbid conditions were flagged including VOC, pulmonary conditions such as ACS, cerebrovascular conditions (stroke), hepatic conditions (gallstones), splenic conditions (splenic sequestration), and other conditions that commonly occurred among SCD patients. Medications and management procedures frequently used for the SCD population were identified during the baseline period. Baseline all-cause HRU were also identified by inpatient, outpatient (emergency room, office, other), and pharmacy visits.

### Outcome Measures

Vaso-occlusive events were defined as an inpatient stay with a primary or secondary clinical claim of SCD with crisis (ICD-9-CM: 282.42, 282.62, 282.64, 282.69) within the SCD population. The event rate of VOC episodes after the index SCD diagnosis was calculated in 100 person-years using the number of events divided by the length of the follow-up period. Deaths were identified by flagging patients who died during the entire follow-up period. The rate of complications was identified for cerebrovascular, hepatic, pulmonary, and splenic conditions. All the clinical events were evaluated separately for pediatric patients (aged <18) and adult patients (aged ≥18). Life-threatening complications including ACS, splenic sequestration, pulmonary embolism, stroke, and pulmonary hypertension were identified by ICD-9-CM diagnosis code.

### Statistical Methods

All variables were first analyzed descriptively. Percentages and numbers were provided for categorical variables. Means and standard deviations were provided for continuous variables.

Cox proportional hazards regression was used for the multivariate analysis of the time to first complication after the index date, concerning the relationship between the rate of follow-up VOC and life-threatening complications requiring acute care—including ACS, splenic sequestration, pulmonary embolism, stroke, pulmonary hypertension, and death. For the Cox model, the rate of follow-up VOC events before the complication and death were identified. Stepwise model selection was applied. Considering the possibility of progression of diseases with time, follow-up VOCs were controlled in the model as time-varying variables. The impact of the complications was also controlled in the model. For example, if stroke is the dependent outcome, then other complications (i.e., ACS, splenic sequestration, pulmonary embolism, pulmonary hypertension) were put in the model. All complications were controlled in the model for the death outcome. Patient demographics and baseline clinical characteristics including age, sex, race, geographic region, CCI scores, baseline SCD management including SCD medication use and SCD management procedures (e.g., blood transfusion and Transcranial Doppler Ultrasonography), and baseline HRU; the number of baseline VOC events were controlled in the model as covariates. Hazard ratios (HR), 95% confidence intervals (CI), and p-values were examined for the follow-up VOC and all other covariates.

### Data Sharing Statement

The data analyzed during the current study are available from the corresponding author on reasonable request.

## Results

### Baseline Characteristics for Overall SCD Patients

**Figure 1.**
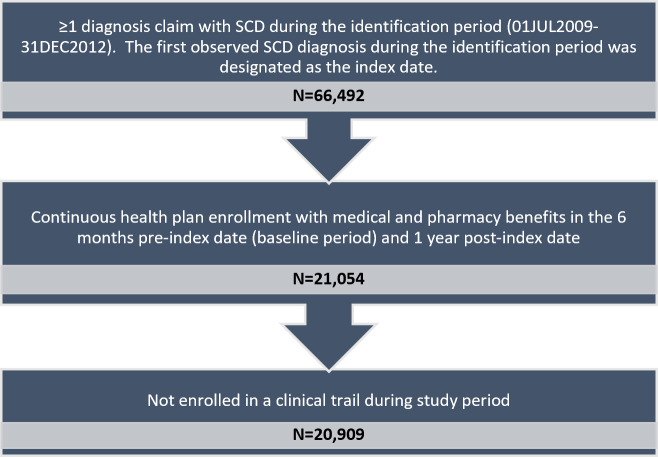
Patient Selection Flow Chart

After applying the inclusion and exclusion criteria, a total of 20909 SCD patients who met the study selection criteria were included (Figure 1).

The mean age of the included SCD patients is 17.94 years old (SD=15.17), and ~60% (59.25%) of the enrolled individuals were pediatric patients (>18 years old). The majority (65.65%) of the SCD patients were African American and had a CCI score of 0 (75.03%). Among all the eligible SCD patients, ~13% (12.82%) had ≥1 VOC requiring inpatient stay during the half-year baseline period. Pulmonary conditions were the most common cause for baseline comorbidities among the enrolled patients: 13.12% of them had upper respiratory tract infections, 11.22% had asthma, and 2.76% had ACS. Other frequent conditions during the baseline period were infectious and parasitic diseases (18.08%) and fever (15.51%). For baseline SCD management, approximately half (49.03%) of the patients were prescribed antibiotics, followed by acetaminophen (35.65%), folic acid (29.19%), and nonsteroidal anti-inflammatory drugs (NSAIDs) (22.86%). During the baseline period, 10.89% of the selected SCD patients had a blood transfusion. For baseline all-cause HRU, 45.50% of the patients had ≥1 outpatient emergency room visit and 23.88% had ≥1 inpatient visit with a mean length of stay of 2.90 days (SD=9.78) (Table 1).

**Table 1.** Baseline Characteristics for Overall SCD Patients

**Patient Characteristics - Population A**	**Sickle Cell patients N=20,909**	
	**N/Mean**	**%/SD**
**Age (years)**	17.94	15.17
<2	1579	7.55%
2-5	3662	17.51%
6-11	3604	17.24%
12-17	3543	16.94%
18-30	4421	21.14%
31-45	2521	12.06%
46-64	1526	7.30%
65+	53	0.25%
**Sex**		
Male	9179	43.90%
Female	11730	56.10%
**Race/Ethnicity**		
White	1448	6.93%
Black	13727	65.65%
Hispanic	2715	12.98%
Other	622	2.97%
Unknown	2397	11.46%
**Geographic Region**		
Northeast	7671	36.69%
North Central	2705	12.94%
South	6715	32.12%
West	3818	18.26%
**Charlson Comorbidity Index Score**	0.42	0.96
0	15688	75.03%
1	3369	16.11%
2-3	1474	7.05%
4+	378	1.81%
**Patients with Baseline VOC**	2680	12.82%
Individual Comorbid Conditions*		
**Pulmonary**		
Upper respiratory tract infections	2744	13.12%
Asthma	2347	11.22%
Acute Chest Syndrome	577	2.76%
Pulmonary embolism	111	0.53%
Pulmonary hypertension	95	0.45%
**Oncologic**		
Neoplasms benign and malignant	926	4.43%
**Cerebrovascular**		
Seizures	647	3.09%
Stroke	342	1.64%
**Spleen**		
Splenic sequestration	112	0.54%
Hypersplenism	20	0.10%
**Others**		
Infectious and parasitic diseases	3781	18.08%
Fever	3242	15.51%
Constipation	1085	5.19%
Iron overload	590	2.82%
Aseptic (Avascular) bone necrosis	445	2.13%
**Baseline SCD Management**		
**SCD Medication**		
Antibiotics	10252	49.03%
Acetaminophen	7455	35.65%
Folic Acid	6103	29.19%
NSAIDs	4780	22.86%
Opioids (Narcotics)	2943	14.08%
Hydroxyurea	1899	9.08%
Iron Chelating therapy	788	3.77%
**Other SCD Management**		
Blood transfusions	2277	10.89%
Transcranial Doppler ultrasonography	1024	4.90%
Pneumococcal vaccine	1020	4.88%
Meningococcal vaccine	226	1.08%
Bone marrow transplants	17	0.08%
**Baseline All-cause Health Care Resource Utilization for all patients (6 months)**		
Any inpatient stay	4993	23.88%
Any outpatient emergency room visit	9513	45.50%
Any outpatient office visit	15342	73.38%
Any outpatient hospital visit	13692	65.48%
Any ambulatory surgery center visit	346	1.65%
Any lab visit	5810	27.79%
Any outpatient other visit	7505	35.89%
Any pharmacy visit	17 205	82.29%

* Baseline Individual comorbidities <1% among overall population were not shown.

### Mortality and Complications among Adults and Pediatric SCD Patients

**Figure 2.**
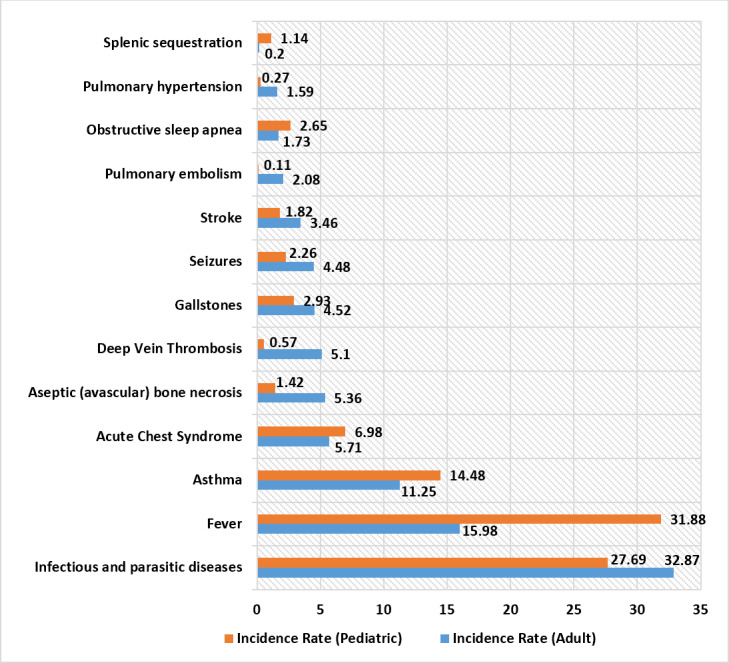
Incidence of Complications in 100 Person-year for Adult and Pediatric SCD Patients

For adult patients, the follow-up mortality rate was 1.06 in 100 person-years. The percentage of adults who developed VOC requiring an inpatient stay during the first 12-month follow-up period was 30.86%; among this percentage of adults with SCD, 74% had >1 VOC events (Table 3). The rate of VOC events in 100 person-years during the entire follow-up period was 142.20 for adults. The five complications with the highest incidence (in 100 person-years) among adult patients were infectious and parasitic diseases (32.87), fever (15.98), asthma (11.25), ACS (5.71), and aseptic bone necrosis (5.36) (Figure 2).

For pediatric patients, the follow-up mortality rate was 0.14 in 100 person-years. The percentage of children who developed VOC requiring an inpatient stay during the first 12-month follow-up period was 21.87%; and among this percentage of children with SCD, 59% had >1 VOC events (Table 3). The rate of VOC events was 53.91 for children in 100 person-years. The five complications with the highest incidence among pediatric patients were fever (31.88), infectious and parasitic diseases (27.69), asthma (14.48), ACS (6.98), and gallstones (2.93) (Figure 2).

**Table 3.** Frequency of VOC during One-year among Medicaid Population

**Frequency of VOC**	**Among All Patients**		**Among Adults with SCD**		**Among Children with SCD**	
	**N**	**Percent**	**N**	**Percent**	**N**	**Percent**
0	15570	74.5%	5891	69.1%	9679	78.1%
1	1789	8.6%	677	8.0%	1112	9.0%
2	1295	6.2%	543	6.4%	752	6.1%
3	584	2.8%	286	3.4%	298	2.4%
4	466	2.2%	257	3.0%	209	1.7%
5	272	1.3%	166	2.0%	106	0.9%
6	238	1.1%	150	1.8%	88	0.7%
7	127	0.6%	99	1.2%	28	0.2%
8	102	0.5%	68	0.8%	34	0.3%
9	66	0.3%	49	0.6%	17	0.1%
10	73	0.4%	53	0.6%	20	0.2%
11	58	0.3%	51	0.6%	7	0.1%
12	48	0.2%	40	0.5%	8	0.1%
13	38	0.2%	31	0.4%	7	0.1%
14	26	0.1%	20	0.2%	6	0.1%
15	17	0.1%	15	0.2%	2	0.0%
>=16	140	0.6%	125	1.4%	15	0.1%

### Relationship between VOC and Life-Threatening Complications for Overall SCD Patients

Cox models were applied to examine the relationship between the frequency of VOCs and clinical endpoints. After the stepwise model selection, significant patient demographics and baseline clinical characteristics remained in the model. Patients who had a follow-up VOC had a 0.55 higher hazard of death than those without a follow-up VOC (95% CI [1.19-2.05]; p value=0.0014). Patients with VOC were also more likely to develop life-threatening complications including ACS (HR=58.67; 95% CI [50.21-68.55]; p value <0.0001), splenic sequestration (HR=34.99; 95% CI [30.65-63.13]; p value<0.0001), pulmonary hypertension (HR=4.12; 95% CI [3.14-5.41]; p value <0.0001), pulmonary embolism (HR=2.82; 95% CI [2.21-3.58]; p value<0.0001), and stroke (HR=2.26; 95% CI [1.94-2.63]; p value <0.0001) (Table 2).

**Table 2.** Cox Model to Examine the Relationship between VOC and Complications

Outcomes among Sickle Cell patients (N=20,909)	Results from Cox Model for Follow-up VOC			
	**HR**	**95% CI**		**p-value**
Time-to-Death^a^	1.56	1.19	2.05	0.0014
Time-to-Acute Chest Syndrome^b^	58.67	50.21	68.55	<0.0001
Time-to-Splenic Sequestration^c^	43.99	30.65	63.13	<0.0001
Time-to-Pulmonary Embolism^d^	2.82	2.21	3.58	<0.0001
Time-to-Stroke^e^	2.26	1.94	2.63	<0.0001
Time-to-Pulmonary hypertension^f^	4.12	3.14	5.41	<0.0001

* Significant covariates after the stepwise model selection for each endpoint model include – ^a^ age, sex, race, region, CCI, baseline neoplasms, baseline VOC, baseline use of opioids, NSAIDs, iron chelating therapy, baseline all-cause HRU, follow-up pulmonary embolism, stroke, and pulmonary hypertension ^b^ age, sex, race, region, baseline use of iron chelating therapy, folic acid, baseline transcranial doppler ultrasonography, baseline all-cause HRU, and follow-up pulmonary embolism ^c^ age, baseline use of hydroxyurea, and baseline pain crisis ^d^ age, sex, race, region, CCI, baseline fever, baseline use of opioids, follow-up acute chest syndrome, stroke, and pulmonary hypertension ^e^ age, sex, race, region, CCI, baseline fever and seizures, baseline use of NSAIDs, iron chelating therapy, tricyclic antidepressants, acetaminophen, baseline blood transfusions and pneumococcal vaccine, baseline VOC, follow-up acute chest syndrome and pulmonary hypertension ^f^ age, CCI, baseline use of opioids, folic acid, baseline blood transfusion, baseline all-cause HRU, follow-up pulmonary embolism, stroke, and acute chest syndrome

## Discussion

This was a retrospective claims study using the US Medicaid database that provides real-world insight into clinical outcomes among the SCD population. Specifically, this study examined the mortality and complication rates for pediatric and adult SCD patients and associations between VOCs and major complications. This study provides a better understanding of SCD’s clinical burden that may provide insight into VOC management among SCD patients.

The rate of pain crisis events in person-years was 1.42 for adults and 0.54 for children in the Medicaid population, which consists of a higher percentage (43%) of children.^21^ In Stettler’s study using the Optum Normative Health Informatics database with a study period of 2009-2013, the VOC rate among adults diagnosed with SCD was 0.7 in one person-year.^22^ The lower number may be due to Stettler’s strict inclusion criteria of VOC that required ≥3 hospitalizations or emergency department visits. Hospitalization rates due to VOC among SCD children and adolescents (0-18 years old) ranged from 0.2 to 1.0 person-years in a large population-based retrospective cohort from 2010 to 2014,^23^ which is consistent with our result when estimating an average value of 0.2-1.0 at nearly 0.6. Besides, the number of VOC events captured in our study may be lower than the real number since only VOC episodes in the inpatient setting were identified to reduce noise and ensure that the disease severity for included patients with VOC was on the same level. The feasibility analysis of VOC prevalence from 2009-2013 (detailed results not shown) indicated that when both outpatient and inpatient VOC events were included, the prevalence rates increased from ~30% to 50% for all years. Additionally, a large proportion of VOC episodes can be managed at home, as VOC without HRU was reported to be ~23% among the 31,017 analyzed patient-days.^6^ Therefore, the rate of actual VOC may be underestimated.

Across all complication-related outcomes, pediatric and adult patients were burdened with multiple complications. Some significant complications with high incidence (in 100 person-years) for both children and adults include infectious and parasitic diseases (27.69 and 32.87), fever (31.88 and 15.98), asthma (14.48 and 11.25), and ACS (6.98 and 5.71). ACS is the leading cause of death among SCD patients according to the Cooperative Study of Sickle Cell Disease. It occurs most frequently among children aged 2-4 years (25.3/100 person-years) and decreases gradually to its lowest value in adults (8.8/100 person-years).^24^ In our study, the ACS rates for both adults and children were lower than expected. While this finding may indicate clinical improvements in the management of ACS in the past two decades, further exploration is required to understand this difference.

After applying a stepwise model selection and adjusting demographics, clinical conditions including baseline cancer diagnosis and baseline VOC requiring inpatient care, SCD management including SCD medication use (opioids, NSAIDs, and iron chelating therapy), and HRU, VOC outcomes in our study were—as hypothesized—significantly associated with death and a number of SCD-related and life-threatening complications including ACS, stroke, pulmonary hypertension, pulmonary embolism, and splenic sequestration. In Platt’s study examining the relationship between VOC and mortality, higher numbers of VOCs were related to higher mortality for those aged >20 years but not related to higher mortality for younger patients.^25^ This discrepancy among age groups in Platt’s study might be due to the smaller sample size of younger patients, as the author suggests. Therefore, the general mortality trend of this study is likely consistent with Platt’s study. The significant relationship between VOC and life-threatening complications is plausible considering the pathological mechanism underlying VOC and all SCD-related complications. Repeated pain episodes can cause bone marrow infarction and necrosis, which lead to pulmonary fat embolism, ACS, and other cardiovascular complications such as stroke and pulmonary hypertension.^26^ Though the exact pathophysiology of how SCD VOC may contribute to further crisis events is not completely clear, previous studies support the evidence that severe pain symptoms often precede such acute events and may be an indicator for special attention for treatment and prevention of further severe complications.^27^

There were also several limitations to this study. While claims data are extremely valuable for the efficient and effective examination of health care outcomes, claims data are collected for payment and not research. First, since VOC events were identified only by using inpatient diagnosis claims, the sample size of patients with VOC might be underestimated since many crisis events can be managed at home; this might reduce the number of VOC and diminish the associations between VOC and complications. In addition, the presence of a diagnosis code on a medical claim cannot confirm the presence of disease, as the diagnosis code may be incorrectly coded or included as rule-out criteria rather than actual disease, which would impact the inclusion of SCD and VOC patients. Second, this study might not be generalizable to other populations. The Medicaid population consists of nearly half of children aged <18 years. This study setting is within the Medicaid population that consists of people with disabilities, low-income children below a certain wage, pregnant women, parents of Medicaid-eligible children who meet certain income requirements, and low-income seniors.^28^ These populations are more likely to have unmet needs in health care resource services. In this study, we excluded individuals with dual eligibility for both Medicaid and Medicare since data of these observations are not complete: due to limited availability, the data of managed care plan patients only include 14 states, and the study period until December 2013 was the most recent data at the time of study. With a mean follow-up time of 2.7 years for all included patients, the mortality rates in our study might not be generalized to long-term mortality and should only be compared to the population with a similar follow-up length.

SCD patients have a substantial burden of complications. Patients experiencing painful crises requiring inpatient care had a significantly higher risk of experiencing subsequent crisis and other life-threatening SCD-related complications. We found strong evidence that VOC is a key risk factor for severe clinical outcomes. Policy-makers and physicians should consider how to prevent severe clinical conditions by improving health care resources and access for those patients with VOCs.

## Authorship Contributions

NS contributed to the interpretation of the data, wrote the manuscript and substantially contributed to critical revisions of the intellectual content.

MB, RH, SA, and JP conceptualized and designed the study, contributed to the interpretation of the data, and substantially contributed to critical revisions of the intellectual content.

LX and HY conceptualized and designed the study, contributed to the acquisition and interpretation of the data, and substantially contributed to critical revisions of the intellectual content.

## Conflict of Interest Disclosures

NS is a consultant and speaker for Novartis. MB, RH, SA, and JP are paid employees of Novartis Pharmaceuticals, the study sponsor. LX is a paid employee of STATinMED Research, a paid consultant to Novartis Pharmaceuticals. HY declares no conflict of interest.
